# Validation and Measurement Invariance of the Leuven Obsessional Intrusions Inventory in Two Different Cultures

**DOI:** 10.5334/pb.537

**Published:** 2020-10-16

**Authors:** Fulya Ozcanli, Laurence Claes, Eva Ceulemans, Dirk Hermans, Batja Mesquita

**Affiliations:** 1 Faculty of Psychology and Educational Sciences, KU Leuven, Leuven, BE; 2 Faculty of Medicine and Health Sciences, University of Antwerp, Antwerp, BE

**Keywords:** Obsessions, intrusive thoughts, OCD, culture, measurement invariance

## Abstract

Obsessions – recurrent unwanted intrusive thoughts – are one of the two pillars of the Obsessive Compulsive Disorder (OCD). Although OCD has been reported across many different cultures, research on these cultural variations is hampered by the lack of cross-culturally sound instruments to assess intrusive thoughts. The aim of the current study is to investigate the psychometric properties of the recently developed Leuven Obsessional Intrusions Instrument (LOII) in two different cultural contexts. Turkish (N = 663) and Belgian (N = 496) participants were sampled from non-clinical student populations. Results from confirmatory factor analyses yielded a shortened version of the LOII (i.e., LOII-R) with a four-factor solution – aggressive, sexual, and contamination intrusions, and ‘just-right’ doubts – as the best fitting model across both cultures. The model met most criteria for strong measurement invariance, and proved to be both valid and reliable. The results of this study suggest that the LOII-R is a good candidate for cross-cultural studies on obsessional intrusions.

## Introduction

Obsessive Compulsive Disorder (OCD) is one of the leading causes of (mental) disability (Torres et al., 2017). It is characterized by recurrent intrusive thoughts, images, or impulses (obsessions), and repetitive, ritualistic behaviors (compulsions) performed to reduce the anxiety triggered by obsessions (American Psychiatric Association, 2013). Obsessions that occur in OCD can be traced back to the unwanted intrusive thoughts, which are experienced by a vast majority of non-clinical populations (Rachman & de Silva, 1978). Intrusive thoughts are assumed to turn into obsessions when the individual attaches significance to them (Rachman, 1997; Salkovskis, 1985). In support of this idea, studies have found that intrusive thoughts reported by non-clinical populations share the themes of obsessions in clinical samples (e.g., Freeston et al., 1991; Purdon & Clark, 1993; Van Oppen et al., 1995), suggesting that research with non-clinical samples might shed light on obsessions (Abramowitz et al., 2014).

Most research on intrusive thoughts has been conducted within Western samples (e.g., Freeston et al., 1991; Moulding et al., 2007; Purdon & Clark, 1993). A small number of studies within non-Western samples exist, but given the lack of cross-culturally sound scales, these studies are not conclusive about the cross-cultural similarities and differences in intrusive thoughts (see for a review of these studies: Ozcanli et al., 2019). As psychological scales developed in one context do not automatically translate to other cultural settings (Fischer & Fontaine, 2010), the development of a cross-culturally sound instrument will be essential for an adequate assessment of cultural variations in intrusive thoughts. The aim of the present study is to develop such instrument, by further investigating the psychometric properties of a recently developed measure of intrusive thoughts, the Leuven Obsessional Intrusions Inventory (LOII; Ozcanli et al., 2019) for non-clinical samples in two different cultures (Belgium and Turkey). The LOII was designed to cover a broad range of intrusions that are representative of clinical obsessions.

## Establishing Psychometric Properties Cross-Culturally

For any scale to be meaningfully used cross-culturally, its psychometric properties should be established across cultures. Traditionally, researchers limited this inquiry to demonstrating good cross-cultural reliability and validity of assessment instruments. Although these components are necessary elements of any sound instrument, they do not guarantee that the construct under investigation has the same structure or meaning across the different groups (Vandenberg & Lance, 2000). The measurement invariance of an instrument across cultures has to be established for it to be used in cross-cultural research (Chen, 2008). Three degrees of measurement invariance have been distinguished: (1) *configural invariance* means that the scale items cross-culturally capture the same dimensions of meaning; (2) *metric invariance* implies that the intervals between the numeric values of the scale have the same meaning across the cultures; and (3) *scalar invariance* means that, in addition to the previous types of invariance, a particular rating on a scale has the same meaning across cultures, and that a higher rating in any culture means that there is more of the construct. Currently, there is no known scale of intrusive thoughts for which all three criteria of measurement invariance have been confirmed. In fact, with one exception, there is no evidence that existing intrusion scales have cross-cultural measurement invariance, as psychometric testing of existing scales has been limited to one particular cultural group only.

### Existing Intrusion Scales

In this section, we discuss scales of intrusion that have shown acceptable psychometric standards in previous research; at a minimum, these scales show divergent validity with other constructs.^1^ To date, the Revised Obsessional Intrusions Inventory (ROII; Purdon & Clark, 1993, 1994) has been the most widely used scale of intrusions. The ROII is designed to assess the frequency of intrusions and their appraisals. Studies analyzing the structural composition of the ROII have often yielded two dimensions, one including aggressive and sexual intrusions, and the other including contamination intrusions, and doubts (Lee & Kwon, 2003; Moulding et al., 2007; Purdon & Clark, 1993). The ROII, however, has some shortcomings. The most important is its limited content representation of intrusions: Aggression, sexuality and dirt/contamination are included in the ROII, but other themes such as religious intrusions, doubts (e.g., the house burning down), and ‘just-right’ doubts (e.g., whether a task is done properly or not) are not. Another limitation is that the divergent validity of the ROII though good in some studies (Purdon & Clark, 1994), is not robust (Purdon & Clark, 1993). A final limitation is that the scale has been developed and tested in a North American context, and that no study has attempted to test the cross-cultural validity of the ROII by means of measurement invariance.

Another scale of intrusions is the INPIOS (Inventario de Pensamientos Intrusos Obsesivos; Garcia-Soriano et al., 2011). This questionnaire builds on the ROII, yet expanded its content coverage. The scale structure has been tested in a Spanish cultural context, and yielded six dimensions consisting of intrusions of aggression, sexuality/immorality, symmetry/order, contamination, doubts, and superstition, which could also be grouped into two higher order dimensions. The scale exhibited good convergent and divergent validity in the Spanish sample. However, the fit indices in confirmatory factor analysis fell below the accepted range, suggesting that the construct validity of the scale is insufficient. This renders it highly unlikely that the structure of the INPIOS could be replicated across cultures. Hence, it is implausible that the scale shows cultural measurement invariance.

Recently, Ozcanli et al. (2019) set out to develop a new obsessional intrusions scale, i.e., the Leuven Obsessional Intrusions Inventory (LOII). The scale was designed to assess the frequency of intrusions. Like INPIOS, the instrument covers the broad range of intrusions that are reported in the clinical context. Ozcanli et al. (2019) tested the cross-cultural equivalence of LOII in two different cultures (Turkey and Belgium) through a series of exploratory factor analyses (EFA). The EFAs yielded two cross-culturally similar dimensions of intrusions. The researchers found initial support for metric invariance of these cross-culturally similar dimensions, suggesting that the scale may be a good candidate for cross-cultural comparisons.

The current study aims to further test the cross-cultural psychometric properties of LOII, as the preliminary analyses yielded promising results regarding the cross-cultural applicability of the instrument. We chose to focus on the LOII over other existing scales of intrusions, because it represented a larger range of content representation of intrusions than the ROII (Revised Obsessional Intrusions Inventory; Purdon & Clark, 1993, 1994), and had better psychometric properties than the INPIOS (Inventario de Pensamientos Intrusos Obsesivos; Garcia-Soriano et al., 2011).

## The Current Study

Similar to the previous research with the LOII (Ozcanli et al., 2019), the current study compared non-clinical samples from Belgium and Turkey. These samples were chosen as examples of a more individualistic and a more collectivist culture, respectively (Hofstede, 2001). First, we aimed to confirm the previous exploratory results using a more robust method; namely, confirmatory factor analyses (CFA). Second, we set out to go beyond metric invariance and establish the scalar invariance, cross-cultural reliability, and cross-cultural validity (i.e., convergent and divergent validity) of the LOII.^2^

## Method

### Participants

The participants of the current study were university students from Istanbul and Izmir, Turkey (TR), and from Leuven, Belgium (BE). The initial sample consisted of 553 students total (N_TR_ = 301, N_BE_ = 252). This sample was used to establish convergent and divergent validity. To achieve adequate statistical power for CFA (Wolf et al., 2013), we recruited additional students from the same universities who only completed the LOII. The CFA and the measurement invariance analyses were conducted on data from a total of 1159 students (N_TR_ = 663, N_BE_ = 496), including the participants used for convergent and divergent validity. The majority of the participants were female students, but the gender ratio was not different across cultural contexts (80.6 % women in Turkey and 83.8 % women in Belgium). On average, Turkish participants were slightly older than the Belgian participants (20.73 ± 2.28 in Turkey, 18.65 ± 2.69 in Belgium). Students received course credit for their participation in the study.

### Measures

*Leuven Obsessional Intrusions Inventory* (LOII; Ozcanli et al., 2019) is a self-report instrument designed to measure the frequency of intrusive thoughts, images, impulses, and doubts. The 50 items represent the most common types of obsessional thoughts reported in the literature, and are inspired by items from the Revised Obsessional Intrusions Inventory (ROII, Purdon & Clark, 1993, 1994), and supplemented with items from commonly used OCD scales that measure a combination of obsessions and compulsions.^3^ Items that originally assessed compulsive behavior were, if possible, rephrased to capture the corresponding obsessional thought (e.g., “I repeatedly check that my doors or windows are locked” was rephrased as “Doubts about leaving doors or windows unlocked”). We added items on religious obsessions and on ‘just-right’ doubts, based on detailed descriptions provided by clinical researchers (e.g., Rachman, 1997, 2003; Summerfeldt, 2007). An initial pool of 60 items was sent to a group of OCD-experts who provided feedback on both substance and wording. Following their suggestions, we merged several of the original items, resulting in a final set of 50 obsessional intrusions items (for a full list of the items of the LOII, together with their original sources, see Table S1, Supplementary Material). Items were presented with the following instruction: “Below you will read descriptions of certain thoughts, images or impulses that many people experience in their daily lives. These thoughts pop up in your mind involuntarily”. Subjects rated the frequency of each “thought” on a five-point scale from 0 “never” to 4 “very often”.

The instrument was developed in English and then translated into Dutch and Turkish simultaneously. We checked the translations in two ways: The items were back-translated from Turkish and Dutch to English, and then from Dutch to Turkish. Differences between the translations were resolved through discussion between the translators.

In order to establish convergent validity, we administered the *Padua Inventory-Revised* (PI-R; Van Oppen et al., 1995), an OCD-scale measuring (a) impulses, (b) washing, (c) checking, (d) rumination, and (e) precision dimensions. The PI-R consists of 41 items to be rated on a five-point scale from 0 (not at all) to 4 (very much). The PI-R has demonstrated good convergent and divergent validity in the Dutch OCD sample for which the scale was developed (Van Oppen et al., 1995). A Turkish validation of the PI-R is available (Beşiroğlu et al., 2005); the exploratory factor analyses (EFA) in a Turkish clinical sample yielded the *same* structure that was found in the original Dutch sample, and it also had satisfactory convergent and divergent validity. In the current study, Cronbach’s alpha coefficients for the subscales ranged from .73 (checking subscale) to .81 (rumination subscale) in the Turkish sample, and from .74 (precision subscale) to .87 (checking subscale) in the Belgian sample.

Divergent validity was established using the *Penn State Worry Questionnaire* (PSWQ; Meyer et al., 1990), a scale of worries. Although obsessions and worries share some common features (such as intrusiveness and a lack of perceived mental control), worries are about real-life problems such as finances or health and obsessions often fall outside of the everyday experiences of individuals (Clark & Purdon, 1995; Turner et al., 1992). The PSWQ consists of 16 items to be rated on a five-point Likert scale ranging from 1 (not at all typical) to 5 (very typical). Previous studies showed that both the Turkish (Yilmaz et al., 2008) and the Dutch (Rijsoort et al., 1999) versions of the PSWQ have good convergent and divergent validity. In the current study, Cronbach’s alpha coefficients of the PSWQ were .90 for the Turkish sample, and .92 for the Belgian sample.

### Procedure

Participants completed the questionnaires online, as past research has suggested that online platforms reduce the likelihood of socially desirable answers to sensitive items such as obsessional intrusions (Henderson et al., 2012). Participants first answered demographic questions on their age, sex, educational status, parent education status, country of origin and religion. They then completed the LOII, PSWQ, and PI-R respectively.

### Analyses

#### Model selection

Our model selection started from two models suggested by the exploratory factor analysis (EFA) in the previous study (Ozcanli et al., 2019). In this study, Turkish and Belgian student samples completed the LOII. EFA were conducted on the total sample first. Next, each culture’s solutions were compared to the factorial solutions for the whole sample by carrying out Procrustes rotations. If the rotation of factorial loadings of each culture towards the loadings of the pooled data (cultures combined) yields Tucker’s congruence values greater than .90, the factors were considered as similar across the cultures, which suggested metric measurement invariance (De Roover et al., 2014; Lorenzo-Seva & Berge, 2006; van de Vijver & Poortinga, 2002).

At the highest level of abstraction, the EFA yielded a two-factor solution. The two factors consisted of *bad-self* obsessions (i.e., repugnant thoughts; Rachman, 1997) and *bad-outcome* obsessions (i.e., reactive intrusions; Lee & Kwon, 2003). *Bad-self* obsessions included intrusive thoughts related to sexuality, aggression and religion/blasphemy; *bad-outcome* obsessions included contamination, harm-related doubts, and ‘just-right’ doubts. As this solution suggested metric invariance on both of the factors (see Table 1), we included the two-factor solution as *Model 1* to be tested with CFA.

**Table 1:** Factorial solutions of the LOII (Ozcanli et al., 2019).

Two-factor-solution	Factor 1 (Bad-self)		Factor 2 (Bad-outcome)									

	TR	BE	TR	BE								
	**.99**	**.97**	**.98**	**.97**								
**Six-factor-solution**	**Factor 1** **(Mixed doubts)**		**Factor 2** **(Sexual/religious)**		**Factor 3** **(Contamination)**		**Factor 4** **(Aggressive)**		**Factor 5** **(Illness)**		**Factor 6** **(Religious doubts)**	

	TR	BE	TR	BE	TR	BE	TR	BE	TR	BE	TR	BE
	**.99**	**.97**	**.99**	**.93**	**.98**	**.96**	**.97**	**.94**	**.91**	.82	**.92**	.31

*Note*: Equivalence values based on Tucker coefficients, for the Turkish and Belgian sample. separately. Bold values (Tucker coefficients > .90) represent the dimensions that, in the respective countries, are congruent with dimensions in the pooled sample.

However, the two-factor model describes obsessions in a broad and abstract way which poorly fits the clinical presentation of obsessions. As shown in Table 1, the six-dimensional solution more closely represents the actual experience of obsessions. Of the six dimensions of the model, four were invariant: (a) aggression, (b) sexuality/religion, (c) contamination, and (d) a mix dimension of doubts consisting of harm-focused doubts and ‘just-right’ experiences. We submitted these four invariant factorial dimensions as *Model 2* to CFA.^4^

The *third model* tested in the CFA was based on the second. This model tested the same four dimensions of Model 2, but only retained the five highest-loading items per dimension as yielded by the EFA (total N of items = 20).^5^

To sum up, Model 1 (50 items) consisted of two factors as described by *bad-self* obsessions (sexual, aggressive, and religious obsessions), and *bad-outcome* obsessions (contamination obsessions and doubts). Model 2 (44 items) consisted of four factors with sexual/religious, aggressive, contamination obsessions, and doubts (a mix dimension of doubts consisting of harm-focused doubts and ‘just-right’ doubts). Finally, Model 3 (20 items) consisted of the five highest loading items in both of the countries for the four factors in Model 2.

CFA’s were conducted for Turkish and Belgian samples separately to test the culture-specific fit of the three different models, using R packages Lavaan 0.5–15 (Rosseel, 2012) and SemTools 0.4–0. To determine how well each model describes the data, we examined the goodness of fit indices in both cultures. We used the diagonally weighted least squares (DWLS) estimator of the goodness of fit, as this approach is often recommended for ordinal indicators with few categories of Likert scales (Rhemtulla et al., 2012). Moreover, the DWLS estimator calculates the robust fit indices by correcting for non-normality, which was the case in our data. Adopting the conventions of the field, a model is considered to have good fit to the data when the comparative fit index (CFI) ≥ .95, and the Root Mean Square Error of Approximation (RMSEA) < .06 (Hu & Bentler, 1999); and an acceptable model fit is considered when CFI > .90, and RMSEA < .08 (MacCallum et al., 1996).^6^ To establish measurement invariance, we selected one model (out of the three) based on model fit.

#### Measurement invariance

For the selected model only, we check measurement invariance in two ways. First, we evaluate the *multigroup* (Turkish vs. Belgian) goodness of fit for every step of measurement invariance (configural, metric, and scalar), using the criteria as described before (CFI, RMSEA). Second, we examine the *change* in the goodness of fit indexes for increasingly stringent equality constraints imposed on the measurement model in every step. Only when the more constrained model is not significantly worse than the less constrained model as indicated by (1) ΔCFI < .01, and (2) ΔRMSEA < .015), we conclude that the next step of measurement invariance has been established as well (Cheung & Rensvold, 2002).

#### Reliability and validity of the LOII

We determine the reliability in terms of the internal consistency of the subscales (by means of Cronbach alpha coefficients). The internal consistency will be defined as good when alpha > .80, and as acceptable when alpha > .70 (George & Mallery, 2003, p. 231). The convergent and divergent validity are evaluated based on the correlations between the scales, separately for the Turkish and Belgian samples. For convergent validity, we expect high correlations (≥.50; Cohen, 1988) both between the LOII (obsessions) and the PI-R (OCD), and between their respective subscales. For divergent validity, we expect low (.10 ≤ r ≤ .29) to moderate (.30 ≤ r ≤ .49) correlations between the LOII (obsessions) and the PSWQ (worry). We also expected low to moderate correlations between the various LOII-subscales and the PSWQ; the PSWQ has no subscales.

To further test divergent validity of the LOII, we compared the correlations between the total scores of the LOII and PI-R with the correlations between the total scores of the LOII with PSWQ, by means of Fisher z test. We expected significantly higher correlations between the LOII (obsessions) and PI-R (OCD) compared to the correlations between the LOII (obsessions) and PSWQ (worry).

## Results

### Model Selection

The results of the confirmatory factor analyses of Models 1, 2 and 3 are shown in Table 2. Model 3, consisting of the dimensions of aggressive, sexual, contamination obsessions, and just-right doubts was the best fitting model in both countries.

**Table 2:** Robust fit indexes of the different models according to DWLS estimator.

	Turkey			Belgium		

	CFI	RMSEA (90% CI)	χ^2^	CFI	RMSEA (90% CI)	χ^2^

Model 1	.822	.070 (.068–.072)	4707.98***	NA	NA	NA
Model 2	.894	.058 (.056–.061)	2773.89***	.924	.057 (.54–.060)	2209.98***
Model 3	.948	.062 (.056–.067)	560.12***	.962	.064 (.057–.071)	476.37***

Models 1 and 2 failed to show cross-culturally good fit. Model 1 showed poor fit in the Turkish sample, and it completely failed to converge in the Belgian sample. Model 2 exhibited acceptable fit in the Turkish. Even though the fit indices suggested good fit for the Belgian sample, the Model 2 caused identification problems.^7^

**Figure 1:**
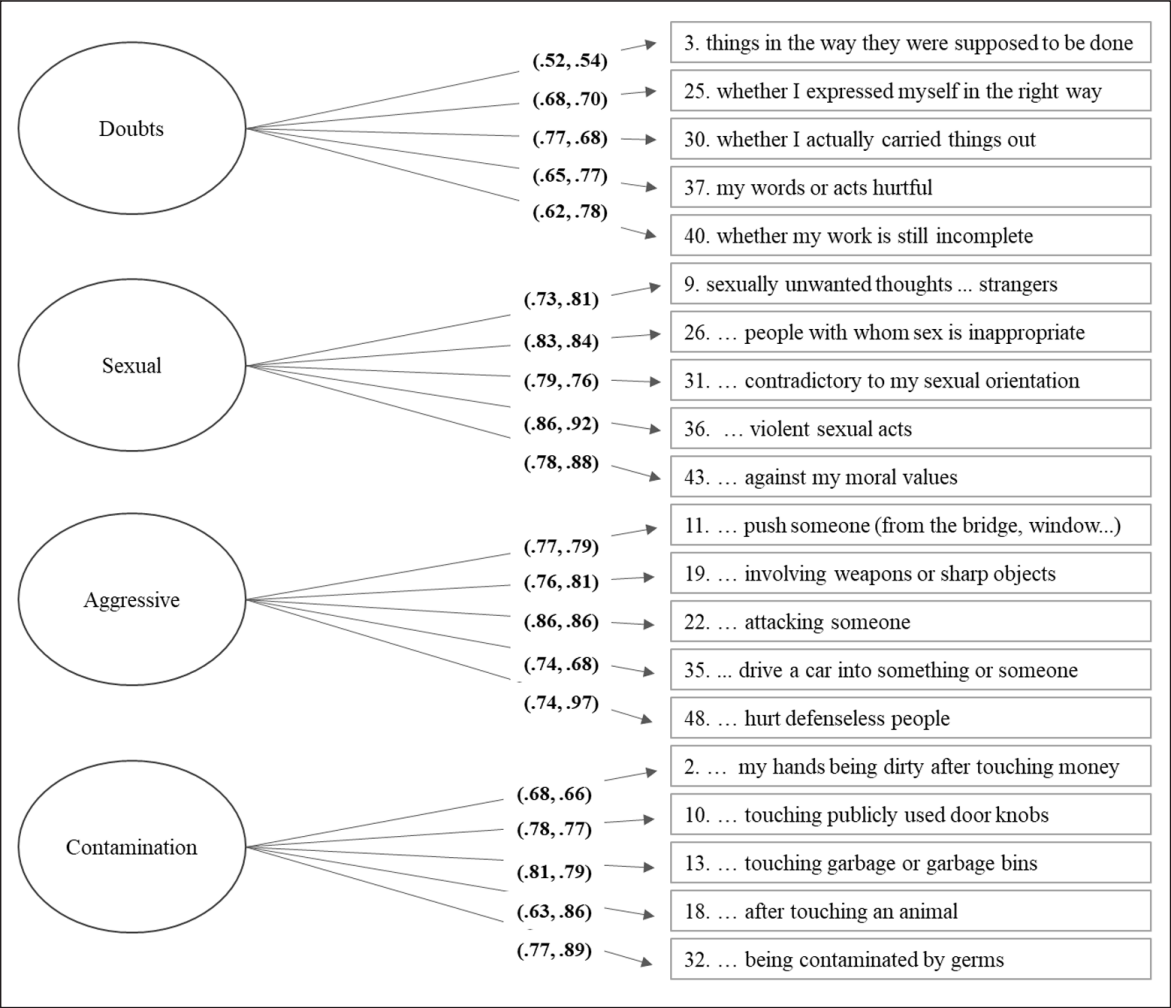
Standardized parameter estimates for Turkish and Belgian LOII-R factor model (Model3). Numbers in parentheses are Turkish followed by Belgian loading estimates; all parameter estimates are significant at *p* < .001.

Based on these results, Model 3 (the 20-item shortened version) was chosen as the best fitting structural model in both Turkey and Belgium. All further analyses will be conducted using this structural model. Figure 1 presents the graphical representation of the model with item loadings in both Turkish and Belgian samples. The 20-items shortened version will be referred to as LOII- revised (LOII-R) from here on.

### The LOII-R: Measurement Invariance

We tested whether the factor structure of LOII-R (Model 3) was invariant across the Turkish and the Belgian group. The fit of each level of invariance is presented in Table 3. Both the configural and the metric models showed good fit (CFA > .95; RMSEA < .06), and the model fit did not significantly worsen from the configural level to the metric level. Hence, we found metric invariance for Model 3. Next, we evaluated the scalar invariance of Model 3. The model showed good fit to the data (CFA = .96; RMSEA = .04). Model fit from the metric to the scalar level did not significantly worsen according to one criterion (ΔRMSEA < .015), but stayed at the threshold for the second criterion (ΔCFI < .01). The model offers some support for scalar invariance in Turkey and Belgium, as two out of three criteria for evaluating a good multigroup fit are met.^8^

**Table 3:** Measurement invariance for Model-3.

	X^2^	ΔX^2^	Df	CFI	ΔCFI	RMSEA	ΔRMSEA

Configural	521.49		328	.982		.033	
Metric	616.64		344	.975		.038	
Configural vs metric		95.15			.007		.005
Scalar	796.78		360	.960		.047	
Metric vs scalar		180.14			.015		.009

### The LOII-R: Reliability and Validity

#### Reliability

As shown in Table 4, the internal consistency, as indicated by the Cronbach alpha coefficients of the four LOII-R scales, were acceptable to good for both cultural groups.

**Table 4:** Cronbach alpha coefficients of the LOII-R total scale score and its subscales.

	Turkey	Belgium

Total score	.84	.88
Sexual	.84	.84
Aggressive	.79	.81
Contamination	.81	.83
Doubts	.74	.79

#### Validity

Consistent with our predictions, separate analyses in the Turkish and Belgian samples (see Table 5), yielded moderately high correlations between the LOII-R (obsessions) and the PI-R (OCD), and between their respective subscales; this suggests cross-culturally good convergent validity of LOII-R. Also consistent with our predictions, the correlations between the LOII-R scales with the PSWQ total scale (worry) were low to moderate in both cultures, with one exception: the correlations between the LOII-R doubts subscale and worry were high in both cultures. The results suggest cross-culturally good divergent validity for the three other subscales. Finally, we checked whether the correlations between the LOII-R total scale score (obsessions) and the PI-R total scale score (OCD) were significantly higher than the correlations between the LOII-R total scale score (obsessions) and the PSWQ (worry). The results confirmed our predictions (Turkish student sample: Fisher’s *z* test = 5.88; *p* < .001), (Belgian student sample: Fisher’s *z* test = 7.88; *p* < .001), indicating further evidence for divergent validity of the LOII-R.

**Table 5:** Zero order correlations between the variables in the Turkish and Belgian samples (upper diagonal represents the correlations in the Turkish sample, the lower diagonal in italics represents the correlations in the Belgian sample).

	LOII-T	LOII-S	LOII-A	LOII-D	LOII-C	PSWQ	PI-T	PI-I	PI-W	PI-C	PI-R	PI-P

LOII-T		.70**	.70**	.68**	.64**	.33**	.71**	.63**	.61**	.65**	.69**	.56**
LOII-S	.80**		.54**	.25**	.14*	.09	.34**	.31**	.31*	.34**	.33**	.32**
LOII-A	.77**	.67**		.31**	.17**	.13*	.49**	.48**	.42**	.46**	.43**	.35**
LOII-D	.74**	.39**	.40**		.33**	.50**	.57**	.48**	.47**	.53**	.54**	.52**
LOII-C	.74**	.46**	.39**	.37**		.18**	.49**	.42**	.47**	.48**	.55**	.34**
PSWQ	.26**	***.1 ***	***.08 ***	.40**	.16*		.47**	.41**	.42**	.47**	.48**	.47**
PI-T	.77**	.63**	.58**	.57**	.59**	.29**		.91**	.93**	.92**	.94**	.88**
PI-I	.63**	.60**	.68**	.31**	.36**	***.11 ***	.70**		.80**	.81**	.84**	.76**
PI-W	.65**	.48**	.41**	.31**	.74**	***.1 ***	.78**	.57**		.84**	.86**	.76**
PI-C	.48**	.37**	.29**	.44**	.35**	.21**	.79**	.31**	.48**		.83**	.79**
PI-R	.69**	.51**	.50**	.69**	.39**	.51**	.83**	.53**	.46**	.58**		.79**
PI-P	.46**	.48**	.36**	.26**	.38**	***.02 ***	.75**	.49**	.56**	.57**	.43**	

*Note*: LOII = Revised Leuven Obsessional Intrusions Inventory, PSWQ = Penn State Worry Questionnaire, and PI = Padua Inventory-Revised, LOII-T = LOII total scale score, LOII-S = LOII Sexual intrusions, LOII-A = LOII Aggressive intrusions, LOII-D = LOII Doubts, LOII-C = LOII Contamination intrusions, PSWQ = PSWQ total score, PI-R-T = Padua Inventory total score, PI-R-I = Padua Inventory Impulses subscale, PI-R-W = Padua Inventory Washing subscale, PI-R-C = Padua Inventory Checking subscale, PI-R-R = Padua Inventory Rumination subscale, and PI-R-P = Padua Inventory Precision subscale. * *p* < .05, ** *p* < .01.

## Discussion

The aim of the current study was to investigate the cross-cultural psychometric properties of the Leuven Obsessional Intrusions Instrument (LOII) in two different cultures (Belgium and Turkey). Building on a previous exploratory study (Ozcanli et al., 2019), we replicated a four-factor model with the dimensions of sexual intrusions, aggressive intrusions, contamination intrusions, and ‘just-right’ doubts by means of confirmatory factor analysis. This model showed good fit in both the Turkish and Belgian samples. Second, we established metric invariance of the LOII-R, and moreover, all but one criterion (i.e., ΔCFI) for scalar invariance were met. It is remarkable that we were able to establish measurement invariance, given that the few studies before that have attempted this, failed to do so. For instance, studies with African American and European American samples failed to establish measurement invariance in scale of obsessions and compulsions (Garnaat & Norton, 2010; Thomas et al., 2000; Williams et al., 2005). To our knowledge, the LOII-R is the first OCD-related scale for which this level of cross-cultural measurement invariance has been established.

Third, the current study also has proven that the LOII-R is a reliable and valid instrument. The four subscales of the LOII-R showed good internal consistency coefficients across samples, suggesting reliability. Additionally, as expected, the LOII-R scales showed moderately high correlations with the PI-R, assessing OC symptoms, suggesting convergent validity. As for divergent validity, the results showed a low correlation between the LOII-R (assessing obsessions) and the PSWQ (assessing worry). Taken together, these findings suggest that LOII-R has adequate validity in non-clinical student samples.

In sum, the good cross-cultural psychometric properties distinguish the LOII-R from the existing intrusions/obsessions measures, which either have not examined psychometric properties of the scale in as much detail (e.g., Garcia-Soriano et al., 2011; Lee & Kwon, 2003), or have not met the same psychometric standards of good quality measures (e.g., Edwards & Dickerson, 1987).

## Limitations

The purpose of this study was to test the cross-cultural applicability of the LOII in a Turkish and Belgian sample. Although we were able to develop a scale for measuring intrusive thoughts across cultures, we also noted some limitations. First, in order to achieve construct equivalence in different cultures we excluded many items that may be culturally meaningful in one or both cultures. Specifically, we removed the dimensions of religious intrusions and harm-related doubts from the item pool.

A second limitation of the present study was that we only established psychometric properties of the LOII-R in non-clinical student samples. Future research should examine the utility of the instrument for clinical groups. Finally, this research tested the cross-cultural psychometric properties of LOII-R only in two cultures. Future research should examine whether the structure we obtained in this study has measurement invariance in other cultural contexts as well.

## Conclusion

Taken together, the results of the current study indicated that LOII-R with its cross-culturally invariant factorial structure, and satisfactory validity evidence is a good candidate as a measure of obsessional intrusions across different cultures, at least among the Belgian and Turkish cultural groups. Importantly, the study shows yet again that an instrument with good psychometric properties in one culture need not have good psychometric properties in another cultural setting. Measurement equivalence across cultures cannot be taken for granted.

## Data Accessibility Statements

Raw data can be found on OSF (https://osf.io/dpgc5/quickfiles).

## Additional File

The additional file for this article can be found as follows:

10.5334/pb.537.s1 ESM 1. Table S1. The LOII items and the original item sources.

## Footnotes

A number of other instruments developed to measure obsessional intrusions (e.g., Clark & de Silva, 1985, Freeston et al., 1991), but the majority of these scales do not meet the minimal psychometric standards even within a single culture (for a review, see Julien et al., 2007), as they fail to show divergent validity with measures of depression, anxiety, or worry. We limit our discussion here to the scales that meet these minimal standards.

New samples were recruited for the current samples. Samples were not overlapping with those of Ozcanli et al, 2019.

We developed the LOII before learning about the INPIOS. Both LOII and INPIOS started from the ROII, and aimed to broaden the content coverage. Using the LOII, we were able to establish a scale with cross-cultural invariance; however, not without dropping lower loading items. It is possible that, using a similar analytic strategy (e.g., recruiting the items with the highest factorial loadings), INPIOS could similarly offer a jumping board for a scale with stronger construct validity (i.e., better fit indices in CFA), and more cultural invariance.

The EFA in Ozcanli et al (2019) also yielded a four and a five-factor model. We chose to start from the 6-factor model over the other two possible models to preserve the distinction between sexual and aggressive obsessions. This seemed important because studies with clinical samples have yielded two separate dimensions (e.g., Calamari et al., 2004; Mataix-Cols et al., 1999).

We also tried to recruit the highest loaded items on the two-factor model, but eventually dropped this model, because the highest loaded items were different in both cultures.

Given that χ2 is highly dependent on sample size, and can lead to the rejection of plausible models, we do not rely on χ2 to select the best model. Nevertheless, χ2 has been reported in Table 2 for descriptive purposes.

We also tested the model fit using robust maximum likelihood (MLR) estimator and evaluated the models based on Akaike’s information criterion (AIC) and Bayesian Information Criterion (BIC) values. The best fitting model is considered to have the lowest AIC and BIC values. The results were consistent with the information yielded by DWLS estimator. Model 3 was the best fitting model with the lowest AIC and BIC values in both Turkey (AIC: 34017.47, BIC: 34311.41) and Belgium (AIC: 20711.40, BIC: 20986.18). Model 2 was the second-best fitting model in both Turkey (AIC: 72134.23, BIC: 72744.86) and in Belgium (AIC: 42925.73, BIC: 43494.03). Model 1 was the worst fitting model in both Turkey (AIC: 82777.39, BIC: 83444.32) and Belgium (AIC: 47638.92, BIC: 48260.09).

We also tested gender invariance of the model within each culture. In both cultures, the proportion of female participants was much higher than the proportion of male participants. Therefore, we matched the group sizes of women and men in each culture by randomly selecting a sub-sample of female participants. The DWLS estimator failed to converge due to small sample sizes in comparison to a large number of parameters. However, using an MLR estimator, we were able to establish metric and scalar gender invariance; in both cultures. The model met two of the three criteria of gender invariance; only the CFI values were below the accepted criteria (Turkey: CFA = .83, ΔCFI = .002, ΔRMSEA = .002; Belgium: CFA = .81, ΔCFI = .004, ΔRMSEA = .001), respectively.
