# Supplementary material for: Validation and Measurement Invariance of the Leuven Obsessional Intrusions Inventory in Two Different Cultures
Source: Psychol Belg. 2020 Oct 16;60(1):347–61. doi: 10.5334/pb.537 (PMC7566505; doi:10.5334/pb.537)
Supplement: ESM 1. Table S1. — The LOII items and the original item sources. [file pb-60-1-537-s1.pdf]

1 Supplementary material

Table S1. The LOII items and the original item sources

**The LOII Items**	**Original Item Source**	**Original source items and their location in the measure**
1. Doubts about leaving a door or window unlocked	VOCI	20. I repeatedly check that my doors or windows are locked…
2. Doubts or images about my hands being dirty after touching money	VOCI	3. I feel dirty after touching money.
3. After completing a task, doubts about whether I did things in the way they were supposed to be done	Developed by the authors**	
4. Doubts about my forgetfulness that will put people around me at risk	Developed by the authors*	
5. Thoughts or images about accidents involving a loved one	VOCI	6. I repeatedly experience the same unwanted thought or image about an accident.
6. An impulse to do inappropriate things in a religious context	Developed by the authors	Inspired by Rachman, 2003, pg.110
7. Doubts or images about catching a disease from public restrooms	VOCI	50. I am afraid to use even well-kept public toilets because I am so concerned about germs.
8. An impulse to swear in public	ROII	25. Swearing in public
9. Sexually unwanted thoughts, images or impulses involving strangers	ROII	42. Strangers naked
10. Doubts or images of contamination by touching publicly used door knobs	ROII	46. Contamination-doors
11. Thoughts, images or impulses to push someone (from the bridge, out of the window, into a running traffic…)	ROII	12. Pushing a stranger-train, car
12. Sexually unwanted thoughts, images or impulses involving defenseless people	Developed by the authors	Rachman, 2003, pg. 15
13. Doubts or images of contamination after touching garbage or garbage bins	VOCI	21. I find it very difficult to touch garbage or garbage bins.
14. An impulse to blurt out obscenities in public	VOCI	28. I am often upset by unwanted thoughts or images of blurting out obscenities or insults in public.
15. Doubts about having an illness of which the existence is not yet known	Developed by the authors	Inspired by Y-BOCS
16. Doubts that I might offend God	Developed by the authors	Inspired by Rachman, 2003, pg.110
17. Thoughts, images or impulses of harming myself or others	VOCI	40. I am often very upset by my unwanted impulses to harm other people

18. Doubts or images of contamination after touching an animal	VOCI	32. I feel very contaminated if I touch an animal
19. Thoughts, images or impulses involving weapons or sharp objects	VOCI	2. I am often upset by my unwanted thoughts of using a sharp weapon
20. Doubts or images about being contaminated, even after slight contact with bodily fluids (sweat, saliva, urine…)	Padua Inventory	2. I think even slight contact with bodily secretions (perspiration, saliva, urine etc.) may contaminate my clothes or somehow harm me.
21. Doubts that objects might be arranged in a wrong way	Developed by the authors**	
22. Thoughts, images or impulses about attacking someone	Developed by the authors	Rachman, 2003, pg. 26
23. Thoughts, images or impulses to steal something	ROII<br>Padua Inventory	32. Shoplifting<br>55. Steal something from the supermarket
24. Doubts about being contaminated without knowing it	ROII	52. Dirt in unseen places
25. After having talked to someone, doubts about whether I expressed myself in the right way	Y-BOCS	33. Fear of not saying just the right thing
26. Sexually unwanted thoughts, images or impulses involving people with whom sex is inappropriate	ROII	35. Sex with an unacceptable person
27. Doubts about accidentally causing harm to other people without knowing it	Y-BOCS	8. Fear of causing an accident without being aware of it
28. Doubts about accidentally hitting a pedestrian while driving	Y-BOCS	8. Hit-and-run automobile accident
29. Inappropriate thoughts or images involving important religious figures (prophet, imam, priest…)	Developed by the authors	Inspired by Rachman, 2003, pg.110
30. After having done things, doubts about whether I actually carried them out	PI-WSUR	27. Sometimes I am not sure I have done things which in fact I know I have done.
31. Unwanted thoughts, images or impulses contradictory to my sexual orientation	ROII	40. Acts against sexual preference
32. Doubts or images about being contaminated by germs	Y-BOCS	12. Concern with dirt or germs
33. An impulse to shout out blasphemous words	Developed by the authors	Inspired by Rachman, 2003, pg.110
34. Doubts about catching a fatal disease (AIDS, Ebola …)	ROII	48. Catching a fatal disease-strangers
35. Thoughts, images or impulses to drive a car into something or someone	Padua Inventory	49. Impulse to drive the car into someone or something
36. Unwanted thoughts, images or impulses involving violent sexual acts	Developed by the authors	Rachman, 2003, pg. 25
37. Doubts that my words or acts will be interpreted as hurtful	Developed by the authors*	
38. Thoughts, images or impulses to	ROII	3. Hitting animals, people

hurt animals		
39. Doubts about skipping important information while reading a book, newspaper or magazine	Developed by the authors**	
40. After being done with a project, doubts of whether my work is still being incomplete	Developed by the authors**	
41. Doubts about my religious faith		Inspired by Rachman, 2003, pg.110
42. Doubts about performing a religious task or ritual in the right way	Developed by the authors	Inspired by Rachman, 2003, pg.110
43. Sexually unwanted thoughts, images or impulses contradictory to my moral values	VOCI	52. I repeatedly experience upsetting and unwanted immoral thoughts
44. A thought, image or an impulse to publicly expose myself	ROII	39. Exposing myself
45. Doubts about being poisoned by chemical substances (household cleaning products, poisonous substances, radiation…)	Y-BOCS	13. Excessive concern with environmental contaminants
46. Doubts about harming others by spreading germs	Y-BOCS	17. Concerned I will get others ill by spreading contaminant
47. Thoughts of acting immorally	VOCI	52. I repeatedly experience upsetting and unwanted immoral thoughts
48. Thoughts, images or impulses to hurt defenseless people	Padua Inventory	56. Impulse to hurt defenseless children or animals
49. Doubts about causing disastrous consequences to loved ones or myself by my being reckless	Developed by the authors*	
50. Doubts about whether I switched off the lights, stove or iron	VOCI	7. I repeatedly check things like taps and switches after turning them off<br>37. I repeatedly check that my stove is turned off

Note. The consulted symptom scales are: The Yale-Brown Obsessive Compulsive Scale (Y-BOCS; Goodman et al.,1989); Revised Obsessional Intrusions Inventory (ROII; Purdon and Clark, 1993, 1994); the Padua Inventory (PI; Sanavio, 1988), the Revised Padua Inventory (PI-WSUR; Burns, Keortge, Formea, and Sternberger, 1996); the Vancouver Obsessional Compulsive Inventory (VOCI; Thordarson et al.,2004); and the Dimensional Obsessive Compulsive Scale (DOCS; Abramowitz et al., 2010).

*Inspired by the clinical accounts of Rachman (2002), the DOCS (Abramowitz et al., 2010), & the “Checking Compulsions” category of Y-BOCS (Goodman et al., 1989).

** Inspired by the items in the “Just-right” dimension of the VOCI (Thordarson et al., 2004), the ‘Repeating Compulsions’ category of Y-BOCS (Goodman et al., 1989), and the clinical descriptions of ‘’Just-right” doubts of Summerfeldt et al. (2007), and Reid et al. (2009).

References for the original item sources of the LOII

Abramowitz, J. S., Deacon, B. J., Olatunji, B. O., Wheaton, M. G., Berman, N. C., Losardo, D., … Hale, L. R. (2010). Assessment of obsessive-compulsive symptom dimensions: Development and evaluation of the dimensional obsessive-compulsive scale. *Psychological Assessment, 22*(1), 180–198. DOI: https://doi.org/10.1037/a0018260.

Burns, G. L., Keortge, S. G., Formea, G. M., & Sternberger, L. G. (1996). Revision of the Padua inventory for obsessive compulsive disorder symptoms: distinctions between worry, obsessions, and compulsions. *Behaviour Research and Therapy, 34*, 163–173. DOI: https://doi.org/10.1016/0005-7967(95)00035-6.

Goodman, W. K., Price, L. H., Rasnysseb, S. A., Nazyre, C., Fleischmann, R. L., Hill, C. L., Heninger, G. R. & Cgarbet, D. S. (1989). The Yale- Brown Obsessive Compulsive Scale. *Archives of General Psychiatry, 46*, 1006-1011. DOI: https://doi.org/10.1001/archpsyc.1989.01810110048007.

Purdon, C., & Clark, D. A. (1993). Obsessive intrusive thoughts in nonclinical subjects. Part I. Content and relation with depressive, anxious and obsessional symptoms. *Behaviour Research and Therapy, 31*, 713–720. DOI: https://doi.org/10.1016/0005-7967(93)90001-B.

Purdon, C., & Clark, D. A. (1994). Obsessive intrusive thoughts in nonclinical subjects. Part II. Cognitive appraisal, emotional response and thought control strategies. *Behaviour Research and Therapy, 32*, 403–410. DOI: https://doi.org/10.1016/0005-7967(94)90003-5.

Rachman, S. (2002). A Cognitive Theory of Compulsive Checking. *Behaviour Research and Therapy, 40*, 625-639. DOI: https://doi.org/10.1016/S0005-7967(01)00028-6.

Rachman, S. (2003). *The treatment of obsessions*. New York: Oxford University Press Inc.

Reid, J., Storch, E., & Lewin, A. (2009). “Just-right’’ OCD Symptoms. International OCD Foundation. Retrieved from http://www.ocfoundation.org.

Sanavio, E. (1988). Obsessions and compulsions: The Padua inventory. *Behaviour Research and Therapy, 26*, 169-177. DOI: https://doi.org/10.1016/0005-7967(88)90116-7.

Summerfeldt, L. J. (2007). Treating incompleteness, ordering, and arranging concerns. In M. M. Antony, C. Purdon, & L. J. Summerfeldt (Eds.), *Psychological treatment of obsessive-compulsive disorder: fundamentals and beyond* (pp. 187-207). Washington, DC: American Psychological Association.

Thordarson, D. S., Radomsky, A. S., Rachman, S., Shafran, R., Sawchuk, C. N., & Hakstian, A. R. (2004). The Vancouver Obsessional Compulsive Inventory (VOCI). *Behaviour Research and Therapy, 42*, 1289– 1314. DOI: https://doi.org/10.1016/j.brat.2003.08.007.
